# Burnout Syndrome Risk in Child and Adolescent Tennis Players and The Role of Adherence to the Mediterranean Diet

**DOI:** 10.3390/ijerph17030929

**Published:** 2020-02-03

**Authors:** Isabel Peraita-Costa, Agustin Llopis-Morales, Salvador Marí-Bauset, Amelia Marí-Sanchis, Salvador Marí-Sanchis, María Morales-Suárez-Varela

**Affiliations:** 1 Unit of Public Health and Environmental Care, Department of Preventive Medicine, University of Valencia, Avda. Vicente Andrés Estellés s/n, 46100 Burjassot, Valencia, Spain; 2 CIBER in Epidemiology and Public Health (CIBERESP), Institute of Health Carlos III, Avda. Monforte de Lemos 3-5, Pabellon 11 Planta 0, 28029 Madrid, Spain; 3 Clinical Nutrition and Dietetics Unit, Navarra Hospital Complex, Calle de Irunlarrea 3, 31008 Pamplona, Navarre, Spain

**Keywords:** Burnout syndrome, Mediterranean diet, nutrition, KIDMED, child, adolescent, athlete, sport, performance, activity

## Abstract

This cross-sectional study examined the impact of adherence to Mediterranean diet on burnout syndrome risk in 94 athletes 8–15 years old. Diet pattern and burnout syndrome risk were assessed through the Athlete Burnout Questionnaire and the KIDMED Questionnaire. 55.3% of girls and 16.1% of boys had a high risk of burnout syndrome and the risk increased with age. Of the 78.7% with low adherence to Mediterranean diet, 31.1% showed no risk of burnout syndrome, 33.8% had a moderate risk, and 35.1% high risk. Of the 21.3% with a high adherence to Mediterranean diet, 35% had no risk of burnout syndrome, 45% had a moderate risk, and 20% had a high risk. Participants with moderate/high burnout syndrome risk were more likely to be girls and spend a higher number of hours watching television or playing video games. There is not enough statistical evidence in this study to reject the independence between the level of adherence to the Mediterranean diet and the risk of burnout syndrome in children, except in the case of daily consumption of fresh or cooked vegetables.

## 1. Introduction

In the last few decades, the physical, psychological and psychosocial benefits of sports have been established beyond doubt [1,2,3,4,5]. In spite of this, if sport is practiced in an obsessive and uncontrolled way, it can become a risk factor for the development of unhealthy lifestyles [6]. The type of sports discipline, the pressure exerted by the coach or teammates, and the influence of the family [7] can influence the increase in that risk. Activity-related stress, whose associated factors are classified in two general categories—activity demands (physical, psychological, social or organizational aspects of the activity requiring sustained physical/psychological effort or skills and therefore associated with certain physiological/psychological costs) and activity resources (functional in achieving work goals, reducing activity demands and the associated costs, stimulating personal growth, learning and development)—can also play a role in the risk of developing burnout syndrome (BS) [7,8,9]. Certain personality traits such as a high degree of empathy, high degree of altruism, low self-esteem, constancy in action, tendency to emotional over-involvement, locus of external control, unrealistic expectations about work, low self-efficacy, and reduced social skills [10,11,12], as well as an excessive concern for body image, perfectionism, impulsivity, competitiveness or tenacity [13], have also been found to play a role in the development of BS. Likewise, sport is often the central element in the lives of athletes, especially those who develop their activity at a competitive level, because it requires high levels of commitment. All this can lead to a high dependence on self-esteem linked to sports performance, training of high expectations, or greater pressure to achieve success. As a consequence, the presence of certain risk factors such as addiction to exercise, substance abuse [14], injuries, eating disorders [15] and burnout syndrome [16] is not infrequent [17,18,19]. 

While most athlete BS studies to date have focused on elite and high performing adult and adolescent athletes [20,21,22,23,24,25,26,27] and studies of BS among amateur athletes are scarce [20,28], the increasing highly competitive nature of youth sports has fueled trends of extensive, early and multifaceted training, early single sport specialization and participation in frequent and numerous competitive events at young ages [29] and amateur sports now share many of the risk factors for BS that are present in work and professional training environments such as high training volumes, high time demands, demanding performance expectations, frequent intense competition, inconsistent coaching practices, little personal control in sport decision making and negative/critical performance evaluations [30,31,32]. Also, athlete burnout is believed to be mainly caused by poor coping behaviors and problem solving skills [26] which may be even more prevalent in children and adolescents who may not have yet developed appropriate coping mechanisms or skills. Children and adolescents are also likely to present some of the personal characteristics considered as risk factors for BS such as perfectionism, need to please others, non-assertiveness, unidimensional self-conceptualization, low self-esteem and high perception of stress or anxiety [30,31,32].

Based on several studies, BS occurs in between 1% and 5% of athletes [33,34], while by sex, it is calculated to affect from 2% to 6% of male athletes and 1% to 9% of female athletes. The results in Spain show a prevalence of 2.77% of BS in the national sports context [35].

Several studies have highlighted the negative nature of the consequences of BS at the cognitive, physiological and behavioral levels, sports withdrawal being one of the most worrying, since athletes experience an interruption in their one-dimensional identity, which they do not know how to manage, established around sports [36,37,38].

The most widely accepted definition of BS in the sport context is the one proposed by Raedeke (1997) [39]. This author adapted to sport the classical conceptualization of Maslach et al. (1981, 1984) [40,41] elaborated in the field of human services that contemplated three dimensions: emotional exhaustion, reduced personal fulfillment and depersonalization [42]. Raedeke (1997) [39] proposed a definition with the following dimensions: physical/emotional exhaustion, reduced sense of achievement and devaluation of sports practice. Raedeke expanded the emotional exhaustion dimension to include physical fatigue from training and competition. The personal fulfillment dimension was adjusted in terms of feelings of inefficacy in relation to sports performance and achievements, called a reduced sense of achievement. Finally, the most drastic change was that posed by Raedeke within the dimension of depersonalization which was replaced by the devaluation of the sport practiced. The depersonalization implies a negative and carefree attitude towards the clients and/or recipients of the service. In sport, the central element is the sport in itself, so this depersonalization could be assimilated to the negative attitude of athletes towards the sport they practice and their participation in it [43].

Demanding workloads may adversely affect performance, as greater effort is required to do a job where demands are high, and produce burnout [8], leading to exhaustion and disengagement. Occupational burnout therefore occurs when the demands of a job exceed the person’s resources to manage these demands [9] and is common in occupations involving direct interactions with people [44,45]. The more widely studied occupational burnout has been associated with negative dietary patterns and intakes in previous studies on a wide variety of professionals [46,47,48,49,50,51,52,53,54]. The association found between BS and emotional [46,54] and uncontrolled eating [46] suggests that the presence of the risk of BS could be important when explaining eating patterns under conditions of stress.

Due to the presence of some of the same external risk factors described above for a work environment associated with BS and the psychological development stage of the studied population, which hinders their ability to adequately cope with possible stressors, it would be expected that BS is present in young amateur athletes. Given that previous studies have associated BS with negative dietary patterns and intakes in a wide variety of professionals and that the conditions that may be related to the association between BS risk and diet pattern may also be found in amateur youth sports, it would be reasonable to expect similar associations between BS and diet pattern to arise in the context of amateur youth athletic practice.

The Mediterranean Diet (MedDiet) is defined as a diet pattern that has been developed for millennia, as a result of the convergence of many cultures, among the peoples who settled on the shores of the Mediterranean Sea [55,56,57]. It is not only a diet pattern, but it is also a social model, a way of behaving at the table, it being common that meals are shared with family and friends, where the ups and downs of the day are discussed and bonds of kinship and friendship and communication are established [55,56,57]. In this study, the authors have considered the MedDiet as the standard, since it has been the dietary pattern traditionally followed in the location this study took place.

The MedDiet is characterized by an intake rich in nuts, fruits and vegetables, whole grains and pulses (mainly as a source of dietary fiber), fish, low-fat dairy, moderate alcohol intake, limited quantities of red meat and sweets, and with olive oil occupying a privileged place, privative of this diet [58,59]. The consumption of fresh and local products, in terms of seasonality, biodiversity, traditional culinary activities and conviviality, as well as the variety of foods, represents the cornerstone of the MedDiet pattern. Cultural and nutritional aspects, jointly with regular engagement in physical activity, are fundamental parts of this comprehensive Mediterranean lifestyle model [60,61].

Several observational, analytical, and experimental studies have highlighted the potential for health benefits provided by the adherence to the MedDiet pattern [56,57,62,63] through the reduction of risk factors for the majority of non-communicable diseases (type 2 diabetes, metabolic syndrome, CVD, certain types of cancer, cognitive decline, depression and mental disorders).

Favorable health outcomes and a better quality of life cannot be attributed to a single macro or micronutrient or a specific phytochemical, but may be attributed to the synergistic combination of a balanced ratio of ω-6 and ω-3 essential fatty acids, high oleic acid content, and high amounts of dietary fiber, as well as antioxidants, phytonutrients (carotenoids, phytosterols, polyphenols, glycosinolates, sulfur compounds, saponins, polyterpenes) and phytic acid, resulting in antioxidant, anti-inflammatory, and antithrombotic properties, preventing lipoperoxidation, improving lipid profile and endothelial function [63,64,65,66,67,68]. In addition, other non-nutritional aspects of the MedDiet, such as its social, cultural, economic and environmental features, including conviviality, sensory stimulation, socialization, biodiversity, and seasonality, have been recognized as aspects that can reinforce the MedDiet’s beneficial effects on wellbeing, quality of life, and health [69,70].

In this context, the specific aim of this study was to investigate the association between adherence to MedDiet and the presence of BS in a group of child and adolescent tennis players between 8 and 15 years of age.

We assessed the hypothesis that a higher adherence to MedDiet may produce a reduction in BS in child and adolescent athletes.

## 2. Methods

We conducted an exploratory cross-sectional study in Valencia (Spain) to assess the role of MedDiet pattern adherence and BS risk in a convenience sample of child and adolescent tennis players. The study was carried out in the second half of 2017.

### 2.1. Participants

This study, utilizing a convenience sample of 94 athletes—56 boys (59.6%) and 38 girls (40.4%)—was conducted in the Tennis Club of Valencia. The inclusion criteria for the study included being a member of this club, being between 8 and 15 years old, being free of injury or diseases at the time of the study and training a minimum of 2 h a day. First, we excluded the families that declined to participate, then we applied the criteria established and later we excluded those who did not attend the study interview appointments or who did not complete the questionnaires properly. In a first appointment, before we started the research, we explained the study to the parents of all the child and adolescent tennis players involved, including the tests that would have taken place and that data would have been kept confidential in line with the Spanish data protection law. Afterwards, parents were contacted individually to obtain their permission to include athletes in the study. If the parents gave verbal consent and the child/adolescent met the selection criteria, an appointment was arranged for BS and MedDiet assessments which were carried out by trained dieticians. Informed written consent was obtained from either both parents or whichever parent was present at the time of the assessment visit before athletes completed the questionnaires. During this same appointment, the parents were interviewed with a structured questionnaire to obtain information on their child’s age, and their birth and medical history.

The study was conducted in accordance with the Declaration of Helsinki, and the protocol received institutional ethical approval.

### 2.2. MedDiet Assessment

Dietary intake was assessed using the KIDMED test (Mediterranean Diet Quality Index in Children and Adolescents) repeatedly validated in Spain [71,72]. The parents had to answer the KIDMED questionnaire composed of 16 questions (Table 1) for children 8–12 years old while 13–15 years olds completed the questionnaires themselves. This test was used to assess the adherence to the MedDiet in children and adolescents. It consists of 16 items, among which there are 12 questions denoting a positive connotation (olive oil, fruits, vegetables, pulses, cereals, pasta or rice, nuts, fish, dairy products, and yoghurt) and 4 questions denoting a negative connotation (of fast food, baked goods, sweets, and skipping breakfast). Questions denoting a positive connotation are scored with +1, while negative connotation questions are scored with −1. According to the KIDMED index, a score of 0–3 reflects poor adherence to the MedDiet, a score of 4–7 describes adherence that needs improvement, and a score of 8–12 indicates good adherence [72,73]. The test exhibited reasonably good validity for assessing the nutrient intakes of children, and we used an ad hoc computer program developed for this purpose. Trained dietitians updated the nutrient data bank using the available information from the food composition tables for Spain.

### 2.3. Burnout Syndrome Assessment

The Athlete Burnout Questionnaire (ABQ) is the most widely used instrument for the measurement of BS in athletes. It was developed by Raedeke (2009) [74]. We used a reduced model of the Spanish version [75,76], composed of 15 items (plus three alternative statements) that measure three dimensions of BS: physical/emotional exhaustion (PEE), reduced sense of accomplishment (RSA), and devaluation of sport (SD) practice (Table 2). A reduced version enables answering in a shorter time, which decreases the adverse effects of longer questionnaires, such as fatigue or lack of motivation. The ABQ was completed in all cases by the child/adolescent with help from the assessment personnel if needed.

Each answer admitted five possible alternatives: “almost never” (+1) “few times” (+2) “Sometimes” (+3) “Often” (+4) “almost always” (+5). The items were stated in such a way that the greater the numerical response of the athlete, the greater the experienced BS, with the exception of items 1, 11 and 15, which were formulated in the opposite direction; the lower the numerical response, the greater the degree of BS.

### 2.4. Statistical Analysis

KIDMED scores were categorized as poor adherence or need to improve when they were less than or equal to seven and good adherence when they were greater than seven. With regard to risk of BS, we categorized all participants into terciles of risk according to the ABQ score (low: ≤ 19.70, moderate: 19.71 to 24.99, and high: ≥ 25).

Contingency tables were constructed to analyze the relationship between sex, age (categorized in 8–10, 11–12 and 13–15 years) and KIDMED scores (in each item and globally) with the risk of BS that they present. The association between the categorical variables was evaluated by the χ2 test (or Fisher’s exact test, as required). In the study of the relationship between the risk of BS and the KIDMED index of children, crude odds ratios (OR) were also calculated with their 95% confidence intervals.

A Student’s t-test was run to compare the age of onset of BS according to KIDMED adherence for each risk range of BS. With dichotomous categorical variables, we compared the KIDMED adherence (poor and needs improvement vs. good) with each risk range of BS (low vs. moderate/high) using contingency tables, odds ratios (ORs), and the χ2 test, Student’s t-test or Fisher’s exact test, as required, to assess statistical significance. Bonferroni corrections were applied to control for multiple comparisons, as appropriate. Non-parametric statistical tests or Shapiro–Wilk tests were used to confirm the assumptions of normality, linearity, homoscedasticity, and independence. Moreover, in order to evaluate the relationship between the risk of BS (low vs. moderate/high) with the sociodemographic characteristics and KIDMED adherence, a multiple logistic regression analysis was performed. The risk of BS (low vs. moderate/high) was considered as a variable response, and the explanatory variables considered were: age (continuous), sex (two categories), level of studies (two categories), mobile use (two categories), television hours and videogames hours (continuous), siblings (two categories), overall KIDMED index, and fresh or cooked vegetables daily (two categories: poor/you need to improve vs. good). Additionally, we calculated the adjusted ORs with 95% CIs. All the p values were two-tailed. Statistically significant associations were considered when *p* < 0.05.

The data were entered into an Excel spreadsheet, using double-data entry to minimize the risk of errors, and were then transferred to SPSS version 19.0 (IBM SPSS, Statistical Package for Social Sciences, Inc., Chicago, IL, USA). All the p values are two tailed and *p* < 0.05 was considered statistically significant.

## 3. Results

Table 3 shows the relationship between gender and age and the BS risk that they present. This analysis revealed that the risks of the girls shifted towards the extremes, with a lower proportion of girls being lower risk, and higher proportion being high risk when compared to their male peers. There is a statistically significant association between sex and the risk of BS (p < 0.001). The main difference is observed in the most extreme, low- and high-risk categories, with the percentages of both sexes in the average level of risk being very similar. The percentage of girls increases as the risk increases, while the opposite happens with boys. Specifically, the percentage of girls at high risk of BS is significantly higher (55.3%) than the percentage of boys (16.1%). On the contrary, the percentage of girls with low BS risk is significantly lower (10.5%) than the percentage of boys with low risk of BS (46.4%). Most of the boys in the study have a low risk of BS, whereas most of the girls in the study have a high risk of BS.

There is no statistically sufficient evidence to reject the independence between the children’s age and the type of risk of BS (p = 0.137). However, it is observed that most of the younger children present a low (40%) or medium (42.5%) risk of BS and, on the contrary, the majority of older children present a high risk of BS (44%).

Table 4 shows no statistically significant differences between the ages of onset of BS and the KIDMED adherence index of children for the groups with low (p = 0.784) and moderate (p = 0.818) risk of BS, while the high risk group presents a significantly later (p = 0.027) age of onset in children with better adherence to the MedDiet.

Table 5 shows the relationship between the KIDMED adhesion index (in each item of the test and overall) and the type of risk of BS (low vs. moderate/high) presented by children. There is not enough statistical evidence to reject the independence between the KIDMED adhesion index and the type of BS risk of children except in the case of the daily consumption of fresh or cooked vegetables. In this case, the percentage of children with a medium/high risk of BS is significantly higher when their KIDMED index is ≤ 7 (poor or in need of improvement) (89.5%) compared to those who have a good KIDMED index of ≥ 8 (63.5%). It is also observed that the percentage of children with a low risk of BS is higher in the case of children who have a good KIDMED index of ≥ 8 (37.3%) with respect to those who have a poor KIDMED index or who need improvement (≤ 7) (10.5%). In terms of OR, the odds of presenting a medium/high risk of BS is five times higher in children with a KIDMED index of ≤ 7 (poor or in need of improvement) for the intake of fresh or cooked vegetables daily compared to children with a good KIDMED index (≥ 8). 

In relation to the KIDMED adherence and comparing only the high risk of BS group (3rd tercile) with the reference group (low risk or 1st tercile), there are significant differences between the group with good (≥ 8), and poor/needs improvement (≤ 7) for fresh and cooked vegetables daily (*p* = 0.004), regular fish (*p* = 0.042), and pasta or rice almost daily (0.019). In the case of the use of olive oil at home (*p* = 0.054), freshly cooked vegetables > 1/day (*p* = 0.059), and sweets and candy (*p* = 0.053), although it does not reach statistical significance, we can say that it suggests an association, or there is a tendency towards significance. In the participants with a low risk of BS, a greater proportion ate breakfast, consumed more vegetables, pasta and rice, and had a greater adherence to the overall KIDMED index. 

Table 6 shows the results of the multiple logistic regression model to study the relationship between the risk of BS and the sociodemographic characteristics and adherence to the MedDiet. It is observed that the probability of presenting a moderate/high risk of BS is greater in girls, with the odds of presenting a moderate/high risk of BS being approximately ten times higher in girls than in boys. As the number of hours of television and video games watched by children increases, the probability of their presenting a moderate/high BS risk increases. The odds of having a moderate/high BS risk are multiplied by approximately seven for each one-hour increase in the number of hours of television and video games. In the logistic regression model, the KIDMED score has incorporated vegetable consumption as an explanatory variable given that it was the only KIDMED item to show statistical differences in the previous analysis. The probability of presenting a moderate/high risk of BS is lower in children with a good KIDMED index score for vegetable consumption. The odds of presenting a moderate/high risk of BS is approximately nine times higher in children with a poor KIDMED score for vegetable consumption when compared to children with a good KIDMED score for vegetable consumption.

## 4. Discussion

There is no consensus among previous research comparing the prevalence of risk of BS. In our study, the participating child and adolescent athletes were classified according to their risk of suffering from BS. Of these, 31.9% had a low risk, 36.2% had a medium risk and 31.9% had a high risk of suffering from BS. The results agree with Garces de los Fayos (1993) [77], in whose study the sample was composed of 33 tennis players whose ages ranged from 11 to 16 years, and with a prevalence of around 35% of the sample that would have suffered, at least once, a high risk of BS. Likewise, Rotella et al. (1991) [78] also reported a high risk prevalence of similar BS (35%). However, Medina Mojena et al., (2002) [10] studying a sample of 40 athletes with an age between 18 and 28 years and sports experience between 5 and 16 years, referred to a prevalence of 10%. Similar results were reported in the studies by Vives Benedicto et al. (2004) [79] and De Francisco et al. (2014) [80], who reported a 8.69% high-risk prevalence in a sample of 230 athletes between 14 and 21 years old, and a sample formed of 442 Spanish athletes, aged between 14 and 29 years, with around 55% at risk of suffering from BS, 28.7% at moderate risk, and 12.4% at high risk, respectively. Equally, Pedrosa et al. (2014) [35] reported a prevalence of 2.7% in a sample of 397 participants, aged 13–64 years, in the Spanish sports context. Sánchez-Alcaraz Martínez et al. (2014) [81], whose sample was composed of 84 tennis players, 58 boys and 26 girls, with ages ranging from 13 to 20 years old, referred to similar data. The results of this research show that 4.8% of the study population has high BS values, values similar to those obtained in the studies by Balaguer et al. (2009) [82] in a sample of 225 young tennis players; or in those of Sierra Llamas et al. (2008) [83], and Gustafsson et al. (2007) [34] in athletes of different modalities.

These differences can be explained by considering that individual athletes (e.g., tennis players) may have higher levels of BS than team sports practitioners or that younger athletes tend to have higher levels of BS [84]. In this study, the first assumption is met; however, in this particular case it is the older athletes that present a higher risk of BS.

Several investigations have been conducted aiming to find any association between external (companions, family, coach, public, or club, among others), and internal stressors (personality factors, or specific traits) and different lifestyle variables such as quantity and quality of physical activity [23,85,86,87], or adherence to a healthy diet [88,89]. Despite previous evidence, unfortunately within sports the possible association between eating behaviors and nutritional intake and BS risk has been given little and non-specific attention. As far as we know, there are no previous studies that have specifically addressed in children and adolescent athletes the association between nutritional intake and the risk of BS.

What foods we choose to consume can be one of the most important factors that influence our mental and physical health [90]. The health benefits of the MedDiet and of physical activity have been documented. Over the last few years, this diet has been recognized for its nutritional composition and effects on human health because it shows an excellent profile of macronutrients and essential micronutrients [65,91]. In this context, some particular deficits of micronutrients or the joint effect of a suboptimal intake [92,93] for several of them could be responsible for the increased risk of psychological disorders, such as BS, among participants who follow low quality dietary patterns. 

## 5. Study Limitations

Although the sample sizes were a relatively small representation of the population of the study, we believe that our study offers strong internal validity given the low attrition rate. We are confident regarding the good quality of the self-reported information used for the questionnaires.

Parents and coaches were very interested in the study and were extensively trained and supported in the completion of the records. All this can compensate for the limitations that the selection of the sample may impose or for external validity generalization. We should also point out that the data for our study were compared with the potential confounders, with all children having a similar place of residence and socioeconomic status and being followed up over the same time period. We believe that these factors may help to compensate for limitations in generalizability and external validity associated with participant selection. Finally, it could be that the KIDMED index score should be different for child and adolescent athletes, although for the moment, no alternative recommendations have been proposed.

## 6. Conclusions

Participants with moderate/high BS risk were more likely to be girls and spend a higher number of hours watching television or playing video games. There is not enough statistical evidence in this study to reject the independence between the level of adherence to the Mediterranean diet and the risk of BS in children, except in the case of daily consumption of fresh or cooked vegetables. An increased risk of BS is likely to be inversely associated with healthier dietary patterns, better nutrient profile, and better eating attitudes. The use of questionnaires that evaluate lifestyles and dietary patterns in child and adolescent athletes can help to add additional information when assessing the risk of BS.

Inadequate intake of some macro and micronutrients could play a role in the development of BS. Those responsible for formulating educational and sports policies, sports doctors, nutritionists and trainers should recommend compliance with high-quality dietary patterns, such as the KIDMED index, thus ensuring adequate nutritional intake that provides an adequate level of different macro and micronutrients for improve the mental and physical health of athletes. More studies are required with representative samples of the target population since inadequate dietary intake and dietary behaviors can adversely affect not only the quality of training, and recovery, but also nutritional status, BS, and general health of the athletes. Finally, the main implications of this study for practice are that coaches and parents should make recommendations on a case-by-case basis after considering a dietary assessment together with a ABQ questionnaire in child and adolescent athletes.

## Figures and Tables

**Table 1:** KIDMED test to assess the Mediterranean diet quality scoring [72].

	Items
+1	Takes a fruit or fruit juice every day
+1	Has a second fruit every day
+1	Has fresh or cooked vegetables regularly once a day
+1	Has fresh or cooked vegetables more than once a day
+1	Consumes fish regularly (at least 2–3 times per week)
−1	Goes more than once a week to a fast-food (hamburger) restaurant
+1	Likes pulses and eats them more than once a week
+1	Consumes pasta or rice almost every day (5 or more times per week)
+1	Has cereals or grains (bread, etc.) for breakfast
+1	Consumes nuts regularly (at least 2–3 times per week)
+1	Uses olive oil at home
−1	Skips breakfast
+1	Has a dairy product for breakfast (yoghurt, milk, etc.)
−1	Has commercially baked goods or pastries for breakfast
+1	Takes two yoghurts and/or some cheese (40 g) daily
−1	Takes sweets and candy several times every day

**Table 2:** Spanish version of the ABQ with 15 items (plus three alternative statements *) [75,76].

Item	Factor	Item Text
1	RSA	En él [deporte] estoy logrando muchas cosas que valen la pena.
2	PEE	El entrenamiento me deja tan cansado/a que me falta energía suficiente para hacer otras cosas.
3	SD	Tengo dudas de si el [deporte] merece todo el tiempo que le dedico.
4	PEE	La práctica del [deporte] me deja demasiado cansado/a.
5	RSA	Creo que no estoy logrando mucho en el [deporte].
6	SD	Mi rendimiento en el [deporte] me importa menos que antes.
7	RSA	Pienso que no estoy rindiendo a mi nivel real en el [deporte].
8	PEE	La práctica del [deporte] me deja mentalmente agotado/a.
9	SD	Creo que no me interesa tanto el [deporte] como antes.
10	PEE	Me siento físicamente agotado/a por él [deporte].
11	SD	Me preocupo menos que antes por triunfar en el [deporte].
12	PEE	Me agotan las exigencias físicas y mentales del [deporte].
13	RSA	Parece que, haga lo que haga, no rindo como debería.
14	RSA	Creo que tengo éxito en el [deporte].
15	SD	Estoy dejando de disfrutar del [deporte].
16 *	PEE	Después de practicar [deporte] me encuentro excesivamente cansado/a.
17 *	SD	El [deporte] no me gusta tanto como antes.
18 *	RSA	Creo que se me da bien el [deporte].
PEE = physical/emotional exhaustion. RSA = reduced sense of accomplishment. SD = sport devaluation. Item translation. * alternative statements.		
Item translation		
1. In [sport] I am accomplishing many things that are worthwhile.		
2. The training leaves me so tired that I lack enough energy to do other things.		
3. I have doubts about whether [sport] deserves all the time I dedicate to it.		
4. The practice of [sport] leaves me too tired.		
5. I think I’m not achieving much in [sport].		
6. My performance in [sport] matters less to me than before.		
7. I think I’m not performing at my real level in [sport].		
8. The practice of [sport] leaves me mentally exhausted.		
9. I think I’m not as interested in [sport] as I used to be.		
10. I feel physically exhausted by the [sport].		
11. I worry less than before to succeed in [sport].		
12. I am exhausted by the physical and mental demands of [sport].		
13. It seems that, whatever I do, I do not perform as I should.		
14. I think I’m successful in [sport].		
15. I’m not enjoying the [sport].		
16. After practicing [sport] I find myself excessively tired.		
17. I do not like [sport] as much as before.		
18. I think I’m good at [sport].		

**Table 3:** Distribution of risk terciles of burnout syndrome according to gender and age.

	Low	Moderate	High	Total	*p*-Value *
	n (%)	n (%)	n (%)	n (%)	
**Gender**					< 0.001
Boys	26 (46.4)	21 (37.5)	9 (16.1)	56 (59.6)	
Girls	4 (10.5)	13 (34.2)	21 (55.3)	38 (40.4)	
**Age**					0.137
8–10 years	16 (40.0)	17 (42.5)	7 (17.5)	40 (42.5)	
11–12 years	7 (24.1)	10 (34.5)	12 (41.4)	29 (30.9)	
13–15 years	7 (28.0)	7 (28.0)	11 (44.0)	25 (26.6)	
**Total**	30 (31.9)	34 (36.2)	30 (31.9)	94 (100)	0.535

*p-value obtained from χ^2^ test. Bonferroni correction for multiple comparisons was applied.

**Table 4:** Mean age according to risk of Burnout syndrome and KIDMED score.

	Age (mean ± SD)	*p*-Value *
**Low**		0.784
≤ 7	10.78 ± 1.999	
≥ 8	11.01 ± 1.732	
**Moderate**		0.816
≤ 7	10.72 ± 1.595	
≥ 8	10.89 ± 1.900	
**High**		0.027
≤ 7	11.62 ± 1.699	
≥ 8	12.50 ± 1.291	
**Total**	11.10 ± 1.778	

*p-value obtained from Student’s t-test.

**Table 5:** KIDMED statistics for total sample (n = 94) and according to risk of burnout syndrome.

	Low	Moderate	High	Total	OR (CI 95 %)	*p*-Value *
	n (%)					
**Fruit or fruit juice daily**						
≤ 7	4 (36.4)	4 (36.4)	3 (27.3)	11(11.7)		
≥ 8	26 (31.3)	30 (36.1)	27 (32.5)	83(88.3)	0.798 (0.214–2.967)	0.737
**Second serving of fruit daily**						
≤ 7	20 (37.0)	20 (37.0)	14 (25.9)	54(57.4)		
≥ 8	10 (25.0)	14 (35.0)	16 (40.0)	40 (42.6)	0.566 (0.229–1.399)	0.218
**Fresh or cooked vegetables daily**						
≤ 7	2 (10.5)	13 (68.4)	4 (21.1)	19 (20.2)		
≥ 8	28 (37.3)	21 (28.8)	26 (34.7)	75 (79.8)	5.064 (1.088–23.575)	0.026
**Fresh or cooked vegetables > 1/day**						
≤ 7	17 (32.7)	14 (26.9)	21 (40.4)	52 (55.3)		
≥ 8	13 (31.0)	20 (47.6)	9 (21.4)	42 (44.7)	0.923 (0.385–2.215)	0.858
**Regular fish consumption (at least 2–3/week)**						
≤ 7	4 (36.4)	2 (18.2)	5 (45.5)	11 (11.7)		
≥ 8	26 (31.3)	32 (38.6)	25 (30.1)	83 (88.3)	0.798 (0.214–2.968)	0.738
**> 1/week fast-food (hamburger) restaurant**						
≤ 7	30 (31.9)	34 (36.2)	30 (31.9)	91 (96.8)		
≥ 8	1 (33.3)	1 (33.3)	1 (33.3)	3 (3.2)	1.067 (0.093–12.229)	0.959
**Pasta or rice almost daily (≥ 5 days/week)**						
≤ 7	4 (18.2)	11 (50.0)	7 (31.8)	22 (23.4)		
≥ 8	26 (36.1)	23 (31.9)	23 (31.9)	72 (76.6)	2.543 (0.777–8.321)	0.116
**Cereal or cereal product for breakfast**						
≤ 7	2 (25.0)	3 (37.5)	3 (37.5)	8 (8.5)		
≥ 8	28 (32.6)	31 (36.0)	27 (31.4)	86 (91.5)	1.448 (0.275–7.637)	0.663
**Regular nut consumption (at least 2–3/week)**						
≤ 7	18 (31.6)	22 (38.6)	17 (29.8)	57 (60.6)		
≥ 8	12 (32.4)	12 (32.4)	13 (35.1)	37 (39.4)	1.040 (0.429–2.523)	0.931
**Use of olive oil at home**						
≤ 7	2 (50.0)	1 (25.0)	1 (25.0)	4 (4.3)		
≥ 8	30 (31.9)	33 (35.2)	31 (32.9)	90 (95.7)	0.469 (0.062–3.489)	0.452
**Skips breakfast**						
≤ 7	32 (34.0)	33 (35.1)	29 (30.9)	88 (93.6)		
≥ 8	3 (50.0)	2 (33.3)	1 (16.7)	6 (6.4)	1.937 (0.369–10.151)	0.429
**Dairy product for breakfast**						
≤ 7	2 (40.0)	1 (20.0)	2 (40.0)	5 (5.3)		
≥ 8	29 (32.6)	33 (37.1)	27 (30.3)	89 (94.7)	0.725 (0.115–4.580)	0.733
**Processed baked goods/pastries for breakfast**						
≤ 7	19 (31.7)	21 (35.0)	20 (33.3)	60 (63.8)		
≥ 8	11 (32.4)	13 (38.2)	10 (29.4)	34 (36.2)	1.032 (0.419–2.541)	0.946
**Two yoghurts and/or 40 g cheese daily**						
≤ 7	24 (36.4)	34 (36.4)	18 (27.3)	66 (70.2)		
≥ 8	6 (22.2)	10 (37.0)	12 (44.0)	28 (29.8)	0.591 (0.212–1.645)	0.313
**Sweets and candy several times a day**						
≤ 7	29 (30.9)	33 (35.1)	32 (34.0)	90 (95.7)		
≥ 8	1 (25.0)	1 (25.0)	2 (50.0)	4 (4.3)	0.747 (0.074–7.490)	0.805
**Total KIDMED index score**						
≤ 7	23 (31.1)	25 (33.8)	26 (35.1)	74 (78.7)		
≥ 8	7 (35.0)	9 (45.0)	4 (20.0)	20 (21.3)	1.194 (0.421–3.386)	0.740

*p-value obtained from χ^2^ or Fisher’s exact test.

**Table 6:** Relationship between burnout syndrome risk and sociodemographic characteristics and adherence to selected KIDMED scores.

	OR (CI 95 %)	*p*-Value
**Age**	0.983 (0.527–1.798)	0.956
**Sex** (Boys)	10.150 (2.458–56.687)	0.003
**Level of parental studies**	2.963 (0.890–11.012)	0.085
**Mobile** (No)	0.778 (0.086–7.172)	0.820
**Hours of television and videogames**	7.607 (2.234–33.986)	0.003
**Number of siblings**	1.113 (0.320–3.798)	0.863
**Fresh or cooked vegetables daily** (≥ 8)	9.489 (1.765–83.258)	0.018
**Total KIDMED** (≥ 8)	2.070 (0.506–9.087)	0.314

Values obtained from adjusted multiple logistic regression model. Lowest category was used as reference category if not specified above.

## References

[1] Eime R.M., Young J.A., Harvey J.T., Charity M.J., Payne W.R. (2013). A Systematic Review of the Psychological and Social Benefits of Participation in Sport for Children and Adolescents: Informing Development of a Conceptual Model of Health through Sport. Int. J. Behav. Nutr. Phys. Act..

[2] Wankel L.M., Berger B.G. (1990). The Psychological and Social Benefits of Sport and Physical Activity. J. Leisure Res..

[3] Bailey R. (2006). Physical Education and Sport in Schools: A Review of Benefits and Outcomes. J. Sch. Health.

[4] Yeh H., Stone J.A., Churchill S.M., Wheat J.S., Brymer E., Davids K. (2016). Physical, Psychological and Emotional Benefits of Green Physical Activity: An Ecological Dynamics Perspective. Sports Med..

[5] Siegenthaler K.L., Gonzalez G.L. (1997). Youth Sports as Serious Leisure: A Critique. J. Sport Soc. Issues.

[6] Gould D., Whitley M.A. (2009). Sources and Consequences of Athletic Burnout among College Athletes. J. Intercoll. Sport.

[7] DeFreese J., Smith A.L. (2013). Teammate Social Support, Burnout, and Self-Determined Motivation in Collegiate Athletes. Psychol. Sport Exerc..

[8] Maslach C., Leiter M. (2014). Burnout in the Workplace. Psychology Serving Humanity: Proceedings of the 30th International Congress of Psychology. West. Psychol..

[9] Schaufeli W.B. (2017). Applying the Job Demands-Resources Model. Organ. Dyn..

[10] Medina Mojena G., García Ucha F.E. (2002). Burnout, Locus De Control Y Deportistas De Alto Rendimiento. Cuadernos de Psicología del Deporte.

[11] Torres Valladares M., Lajo R. (2008). Variables Psicológicas Implicadas En El Desempeño Laboral Docente. Revista de Investigación en Psicología.

[12] Moriana Elvira J.A., Herruzo Cabrera J. (2004). Estrés Y Burnout En Profesores. Int. J. Clin. Health Psychol..

[13] Dosil J., González Oya J., Dosil J. (2008). Eating disorders and the athlete’s environment. Eating Disorders in Athletes.

[14] Rice S.M., Purcell R., De Silva S., Mawren D., McGorry P.D., Parker A.G. (2016). The Mental Health of Elite Athletes: A Narrative Systematic Review. Sports Med..

[15] De Bruin A.K. (2017). Athletes with Eating Disorder Symptomatology, a Specific Population with Specific Needs. Curr. Opin. Psychol..

[16] Gustafsson H., Hill A.P., Stenling A., Wagnsson S. (2016). Profiles of Perfectionism, Parental Climate, and Burnout among Competitive Junior Athletes. Scand. J. Med. Sci. Sports.

[17] Lemyre P., Hall H., Roberts G. (2008). A Social Cognitive Approach to Burnout in Elite Athletes. Scand. J. Med. Sci. Sports.

[18] Martin E.M., Horn T.S. (2013). The Role of Athletic Identity and Passion in Predicting Burnout in Adolescent Female Athletes. Sport Psychol..

[19] Sorkkila M., Aunola K., Ryba T.V. (2017). A Person-Oriented Approach to Sport and School Burnout in Adolescent Student-Athletes: The Role of Individual and Parental Expectations. Psychol. Sport Exerc..

[20] Cresswell S.L., Eklund R.C. (2005). Motivation and Burnout among Top Amateur Rugby Players. Med. Sci. Sports Exerc..

[21] Cresswell S.L., Eklund R.C. (2005). Motivation and Burnout in Professional Rugby Players. Res. Q. Exerc. Sport.

[22] DeFreese J., Raedeke T.D., Smith A.L., Williams J.M., Krane V. (2014). Athlete Burnout: An Individual and Organizational Phenomenon. Applied Sport Psychology: Personal Growth to Peak Performance.

[23] Goodger K., Gorely T., Lavallee D., Harwood C. (2007). Burnout in Sport: A Systematic Review. Sport Psychol..

[24] Lavallee D., Goodger K., Gorely T., Harwood C., Williams J.M. (2005). Burnout in Sport: Understanding the Process-from Early Warning Signs to Individualized Intervention. Applied Sport Psychology: Personal Growth to Peak Performance.

[25] Wylleman P., Lavallee D., Weiss M. (2004). A Developmental Perspective on Transitions Faced by Athletes. Developmental sport and exercise psychology: A lifespan perspective. Developmental Sport and Exercise Psychology: A Lifespan Perspective.

[26] Raedeke T.D., Smith A.L. (2004). Coping Resources and Athlete Burnout: An Examination of Stress Mediated and Moderation Hypotheses. J. Sport Exerc. Psychol..

[27] Martínez-Alvarado J.R., Guillén García F., Feltz D. (2016). Athletes’ Motivational Needs regarding Burnout and Engagement. Revista de Psicologia del Deporte.

[28] Tukaiev S., Dolgova O., Lysenko O., Fedorchuk S., Havrylets Y., Rizun V., Vasheka T., van den Tol A.J.M. (2019). Amateur Sport and Emotional Burnout Formation in High School Students. Contemp. Educ. Res. J..

[29] DiFiori J.P., Benjamin H.J., Brenner J.S., Gregory A., Jayanthi N., Landry G.L., Luke A. (2014). Overuse Injuries and Burnout in Youth Sports: A Position Statement from the American Medical Society for Sports Medicine. Br. J. Sports Med..

[30] Malina R.M. (2010). Early Sport Specialization: Roots, Effectiveness, Risks. Curr. Sports Med. Rep..

[31] Faigenbaum A.D., Kraemer W.J., Blimkie C.J., Jeffreys I., Micheli L.J., Nitka M., Rowland T.W. (2009). Youth Resistance Training: Updated Position Statement Paper from the National Strength and Conditioning Association. J. Strength Cond. Res..

[32] Matos N.F., Winsley R.J., Williams C.A. (2011). Prevalence of Nonfunctional Overreaching/Overtraining in Young English Athletes. Med. Sci. Sports Exerc..

[33] Gustafsson H., Kenttä G., Hassmén P. (2011). Athlete Burnout: An Integrated Model and Future Research Directions. Int. Rev. Sport Exerci. Psychol..

[34] Gustafsson H., Kenttä G., Hassmén P., Lundqvist C. (2007). Prevalence of Burnout in Competitive Adolescent Athletes. Sport Psychol..

[35] Pedrosa I., García-Cueto E. (2014). Estudio Del Síndrome De Burnout En Deportistas: Prevalencia Y Relación Con El Esquema Corporal. Universitas Psychologica.

[36] Gustafsson H., Hassmén P., Kenttä G., Johansson M. (2008). A Qualitative Analysis of Burnout in Elite Swedish Athletes. Psychol. Sport Exerc..

[37] Gustafsson H., DeFreese J., Madigan D.J. (2017). Athlete Burnout: Review and Recommendations. Curr. Opin. Psychol..

[38] Hemmatinezhad M., Benar N., Hashemi M., Moemeni S. (2013). The Causes of Career Termination from Sport and their Relationship to Post-Retirement Difficult among Professional Athletes in Iran. Int. J. Sport Stud..

[39] Raedeke T. (1997). Is Athlete Burnout More than just Stress? A Sport Commitment Perspective. J. Sport Exerc. Psychol..

[40] Maslach C., Jackson S.E., Oskamp S. (1984). Burnout in Organizational Settings. Applied Social Psychology Annual: Applications in Organizational Settings: V. 5.

[41] Maslach C., Jackson S.E. (1981). Maslach Burnout Inventory.

[42] Maslach C. (2003). Burnout: The Cost of Caring.

[43] Raedeke T., Smith A., Kentta G., Arce C., De Francisco C., Rui A., Resende R., Albuquerque A. (2014). Burnout in Sport: From Theory to Intervention. Positive Human Functioning from a Multidimensional Perspective. Promoting Stress Adaptation Volume 1.

[44] Schaufeli W.B., Salanova M., Peeters M.C., De Jonge J., Taris T.W. (2014). Burnout, boredom and engagement at the workplace. An Introduction to Contemporary Work Psychology.

[45] Childs J.H., Stoeber J. (2012). Do You Want Me to be Perfect? Two Longitudinal Studies on Socially Prescribed Perfectionism, Stress and Burnout in the Workplace. Work Stress.

[46] Nevanperä N.J., Hopsu L., Kuosma E., Ukkola O., Uitti J., Laitinen J.H. (2012). Occupational Burnout, Eating Behavior, and Weight among Working Women. Am. J. Clin. Nutr..

[47] Stewart-Knox B.J. (2014). Eating and Stress at Work: The Need for Public Health Promotion Intervention and an Opportunity for Food Product Development?. Trends Food Sci. Technol..

[48] Díaz F.C. (2018). Afectación Del Sindrome De Estar Quemado O Burnout En Los Docentes. Medidas Preventivas Y De Intervención Actuales. Revista de Innovación Didáctica.

[49] Chui H., Bryant E., Sarabia C., Maskeen S., Stewart-Knox B. (2019). Burnout, Eating Behaviour Traits and Dietary Patterns. Br. Food J..

[50] Okumus B., Chaulagain S., Giritlioglu I. (2019). Examining the Impacts of Job Stress and Job Satisfaction on Hotel Employees’ Eating Behavior. J. Hosp. Mark. Manag..

[51] Robaina Palmés F., Flores Robaina N., Jenaro Río C., Cruz Ortiz M., Avram E. (2010). Síndrome De Burnout Y Hábitos Alimenticios En Profesores De Enseñanza Secundaria. El Guiniguada. Revista de Investigaciones y Experiencias en Ciencias de la Educación.

[52] Bradfield R.H., Fones D.M. (1984). Recipe for Burnout: The Special Education Teachers’ Diet. Acad. Ther..

[53] Alexandrova-Karamanova A., Todorova I., Montgomery A., Panagopoulou E., Costa P., Baban A., Davas A., Milosevic M., Mijakoski D. (2016). Burnout and Health Behaviors in Health Professionals from Seven European Countries. Int. Arch. Occup. Environ. Health.

[54] Kristanto T., Chen W.S., Thoo Y.Y. (2016). Academic Burnout and Eating Disorder among Students in Monash University Malaysia. Eat. Behav..

[55] Keys A., Keys M. (1975). How to Eat Well and Stay Well the Mediterranean Way.

[56] Keys A. (1980). Seven Countries. A Multivariate Analisys Od Death and Coronary Heart Disease.

[57] Keys A., Mienotti A., Karvonen M.J., Aravanis C., Blackburn H., Buzina R., Djordjevic B., Dontas A., Fidanza F., Keys M.H. (1986). The Diet and 15-Year Death Rate in the Seven Countries Study. Am. J. Epidemiol..

[58] Simopoulos A., Visioli F. (2000). Mediterranean Diets.

[59] Sánchez Villegas A., Zazpe I., Sánchez Villegas A., Sanchez-Taínta A. (2017). A Healthy-Eating Model Called Mediterranean Diet. The Prevention of Cardiovascular Disease through the Mediterranean Diet.

[60] Trichopoulou A., Lagiou P. (1997). Healthy Traditional Mediterranean Diet: An Expression of Culture, History, and Lifestyle. Nutr. Rev..

[61] Donini L.M., Serra-Majem L., Bulló M., Gil Á., Salas-Salvadó J. (2015). The Mediterranean Diet: Culture, Health and Science. Br. J. Nutr..

[62] Estruch R., Ros E., Salas-Salvado J., Covas M.I., Corella D., Aros F., Gomez-Gracia E., Ruiz-Gutierrez V., Fiol M., Lapetra J. (2013). Primary Prevention of Cardiovascular Disease with a Mediterranean Diet. N. Engl. J. Med..

[63] Martinez-Gonzalez M.A., Bes-Rastrollo M., Serra-Majem L., Lairon D., Estruch R., Trichopoulou A. (2009). Mediterranean Food Pattern and the Primary Prevention of Chronic Disease: Recent Developments. Nutr. Rev..

[64] Henríquez Sánchez P., Ruano C., De Irala J., Ruiz-Canela M., Martínez-González M., Sánchez-Villegas A. (2012). Adherence to the Mediterranean Diet and Quality of Life in the SUN Project. Eur. J. Clin. Nutr..

[65] Trichopoulou A., Martínez-González M.A., Tong T.Y., Forouhi N.G., Khandelwal S., Prabhakaran D., Mozaffarian D., de Lorgeril M. (2014). Definitions and Potential Health Benefits of the Mediterranean Diet: Views from Experts Around the World. BMC Med..

[66] Billingsley H.E., Carbone S. (2018). The Antioxidant Potential of the Mediterranean Diet in Patients at High Cardiovascular Risk: An in-Depth Review of the PREDIMED. Nutr. Diabetes.

[67] Bach-Faig A., Berry E.M., Lairon D., Reguant J., Trichopoulou A., Dernini S., Medina F.X., Battino M., Belahsen R., Miranda G. (2011). Mediterranean Diet Pyramid Today. Science and Cultural Updates. Public Health Nutr..

[68] Serra-Majem L., Ortiz-Andrellucchi A., Sanchez-Villegas A., Ferranti P., Berry E., Jock A. (2018). Mediterranean Diet. Encyclopedia of Food Security and Sustainability. General and Global Situation.

[69] UNESCO Intangible Cultural Heritage Mediterranean Diet 2013. https://ich.unesco.org/en/RL/mediterranean-diet-00884.

[70] Hachem F., Capone R., Yannakoulia M., Dernini S., Hwalla N., Kalaitzidis C., International Centre for Advanced Mediterranean Agronomic Studies (CIHEAM) and Food and Agriculture Organization of the United Nations (FAO) (2016). The Mediterranean diet: A sustainable consumption pattern. Mediterra.

[71] Serra-Majem L., Ribas L., García A., Pérez-Rodrigo C., Aranceta J. (2003). Nutrient Adequacy and Mediterranean Diet in Spanish School Children and Adolescents. Eur. J. Clin. Nutr..

[72] Serra-Majem L., Ribas L., Ngo J., Ortega R.M., Garcia A., Perez-Rodrigo C., Aranceta J. (2004). Food, Youth and the Mediterranean Diet in Spain. Development of KIDMED, Mediterranean Diet Quality Index in Children and Adolescents. Public Health Nutr..

[73] Štefan L., Prosoli R., Juranko D., Čule M., Milinović I., Novak D., Sporiš G. (2017). The Reliability of the Mediterranean Diet Quality Index (KIDMED) Questionnaire. Nutrients.

[74] Raedeke T., Smith A. (2009). The Athlete Burnout Questionnaire Manual.

[75] Arce C., De Francisco C., Andrade E., Seoane G., Raedeke T. (2012). Adaptation of the Athlete Burnout Questionnaire in a Spanish Sample of Athletes. Span. J. Psychol..

[76] De Francisco Palacios C. (2015). Reduced Version of the Athlete Burnout Questionnaire (Abq): Preliminary Psychometric Properties. J. Sports Psychol..

[77] Garcés de Los Fayos E.J. (1993). Frecuencia De Burnout En Deportistas Jóvenes: Estudio Exploratorio. Revista de Psicología del Deporte.

[78] Rotella R.J., Hanson T., Coop R.H. (1991). Burnout in Youth Sports. Elem. Sch. J..

[79] Vives Benedicto L., Garcés de Los Fayos E.J. (2004). Incidencia Del Síndrome De Burnout En El Perfil Cognitivo En Jóvenes Deportistas De Alto Rendimiento. Cuadernos de Psicología del Deporte.

[80] De Francisco C., de Los Fayos E.J.G., Arce C. (2014). Burnout En Deportistas: Prevalencia Del Síndrome a Través De Dos Medidas. Cuadernos de Psicología del Deporte.

[81] Sánchez-Alcaraz Martínez B.J., Gómez-Mármol A. (2014). Prevalencia Del Síndrome De Burnout En Tenistas Según Su Orientación Motivacional. Revista Iberoamericana de Psicologí¬a del Ejercicio y Deporte.

[82] Balaguer I., Duda J.L., Moreno Y., Crespo M. (2009). Interacciones Entre Las Perspectivas Situacionales Y Disposicionales De Meta Y El Burnout Psicológico De Los Tenistas Junior De La Élite Internacional [Interplays between Situational and Dispositional Goals Perspectives and Psychological Burnout.]. Acción Psicológica.

[83] Sierra Llamas C., Abello R. (2008). Burnout Y Pensamientos Irracionales En Deportistas De Alto Rendimiento. Psychologia. Avances de la Disciplina.

[84] Olivares Tenza E., Garcés De Los Fayos E.J., Ortín Montero F., De Francisco Palacios C. (2018). Prevalencia De Burnout a Través De Dos Medidas Y Su Relación Con Variables Sociodeportivas. Universitas Psychologica.

[85] Gould D., Tuffey S., Udry E., Loehr J. (1996). Burnout in Competitive Junior Tennis Players: I. A Quantitative Psychological Assessment. Sport Psychol..

[86] Hall P.A., Fong G.T., Epp L.J. (2014). Cognitive and Personality Factors in the Prediction of Health Behaviors: An Examination of Total, Direct and Indirect Effects. J. Behav. Med..

[87] Gould D., Tuffey S., Udry E., Loehr J. (1996). Burnout in Competitive Junior Tennis Players: II. Qualitative Analysis. Sport Psychol..

[88] Jurado D., Burgos-Garrido E., Diaz F.J., Martínez-Ortega J.M., Gurpegui M. (2012). Adherence to the Mediterranean Dietary Pattern and Personality in Patients Attending a Primary Health Center. J. Acad. Nutr. Diet.

[89] Mõttus R., Realo A., Allik J., Deary I.J., Esko T., Metspalu A. (2012). Personality Traits and Eating Habits in a Large Sample of Estonians. Health Psychol..

[90] Rodriguez N.R., DiMarco N.M., Langley S., American Dietetic Association, Dietitians of Canada, American College of Sports Medicine: Nutrition and Athletic Performance (2009). Position of the American Dietetic Association, Dietitians of Canada, and the American College of Sports Medicine: Nutrition and Athletic Performance. J. Am. Diet. Assoc..

[91] Zazpe I., Sánchez-Taínta A., Santiago S., de la Fuente-Arrillaga C., Bes-Rastrollo M., Martínez J.A., Martínez-González M.Á. (2014). Association between Dietary Carbohydrate Intake Quality and Micronutrient Intake Adequacy in a Mediterranean Cohort: The SUN (Seguimiento Universidad De Navarra) Project. Br. J. Nutr..

[92] Serra-Majem L., Bes-Rastrollo M., Román-Vinas B., Pfrimer K., Sánchez-Villegas A., Martínez-González M.A. (2009). Dietary Patterns and Nutritional Adequacy in a Mediterranean Country. Br. J. Nutr..

[93] Trichopoulou A., Costacou T., Bamia C., Trichopoulos D. (2003). Adherence to a Mediterranean Diet and Survival in a Greek Population. N. Engl. J. Med..

